# Molecular Mechanisms Underlying Yatein-Induced Cell-Cycle Arrest and Microtubule Destabilization in Human Lung Adenocarcinoma Cells

**DOI:** 10.3390/cancers11091384

**Published:** 2019-09-17

**Authors:** Shang-Tse Ho, Chi-Chen Lin, Yu-Tang Tung, Jyh-Horng Wu

**Affiliations:** 1 Department of Forestry, National Chung Hsing University, Taichung 402, Taiwan; s741129@hotmail.com; 2 Agricultural Biotechnology Research Center, Academia Sinica, Taipei 115, Taiwan; 3 Institute of Biomedical Science, National Chung Hsing University, Taichung 402, Taiwan; lincc@nchu.edu.tw; 4 Graduate Institute of Metabolism and Obesity Sciences, Taipei Medical University, Taipei 110, Taiwan; 5 Nutrition Research Center, Taipei Medical University Hospital, Taipei 110, Taiwan; 6 Cell Physiology and Molecular Image Research Center, Wan Fang Hospital, Taipei Medical University, Taipei 116, Taiwan

**Keywords:** *Calocedrus formosana*, lung cancer, yatein, cell-cycle arrest, xenograft

## Abstract

Yatein is an antitumor agent isolated from *Calocedrus formosana* Florin leaves extract. In our previous study, we found that yatein inhibited the growth of human lung adenocarcinoma A549 and CL1-5 cells by inducing intrinsic and extrinsic apoptotic pathways. To further uncover the effects and mechanisms of yatein-induced inhibition on A549 and CL1-5 cell growth, we evaluated yatein-mediated antitumor activity in vivo and the regulatory effects of yatein on cell-cycle progression and microtubule dynamics. Flow cytometry and western blotting revealed that yatein induces G_2_/M arrest in A549 and CL1-5 cells. Yatein also destabilized microtubules and interfered with microtubule dynamics in the two cell lines. Furthermore, we evaluated the antitumor activity of yatein in vivo using a xenograft mouse model and found that yatein treatment altered cyclin B/Cdc2 complex expression and significantly inhibited tumor growth. Taken together, our results suggested that yatein effectively inhibited the growth of A549 and CL1-5 cells possibly by disrupting cell-cycle progression and microtubule dynamics.

## 1. Introduction

Natural products have been used for treating disease for thousands of years. More recently, natural products are being continuously developed for pharmaceutical applications, particularly for anticancer, antibacterial, and antiviral applications [1]. To date, explorative and mechanistic studies on new bioactive compounds are still ongoing.

Cells routinely come in contact with endogenous and exogenous stress stimuli, such as toxic chemicals, UV radiation, and reactive oxygen species. These stress stimuli damage chromosomes, affecting DNA replication and chromosome segregation [2]. Each phase of the cell cycle, including G_0_, G_1_, S, G_2_, and M phase, functions to maintain genetic material duplication and cell division. Abnormal expression of cell-cycle proteins disrupts the cell cycle and is thus closely associated with tumorigenesis [3]. Many natural products exhibit anticancer properties by interacting with cell-cycle proteins [4]. Thus, the identification of a new cell-cycle–targeting natural substance may provide new alternatives in cancer chemotherapy.

*Calocedrus formosana* is a valuable softwood species in Taiwan, which not only has high industrial economic value but also exhibits multiple bioactivities [5,6,7,8,9,10,11,12,13,14]. We previously found that the *C. formosana* extract and its active phytocompound, yatein, inhibited the growth of human lung adenocarcinoma A549 and CL1-5 cells by inducing caspase-related apoptosis [15]. However, whether yatein regulates the cell cycle in human lung adenocarcinoma remains unclear. To uncover the mechanisms of yatein-mediated human lung adenocarcinoma growth inhibition, we examined the effects of yatein on cell-cycle progression, tubulin dynamics, and in vivo tumor growth.

## 2. Results

### 2.1. Yatein Induces Cell-Cycle Arrest at G_2_/M Phase and Enhances G_2_/M Phase-Related Protein Expression in Human A549 and CL1-5 Cells

**Figure 1:**
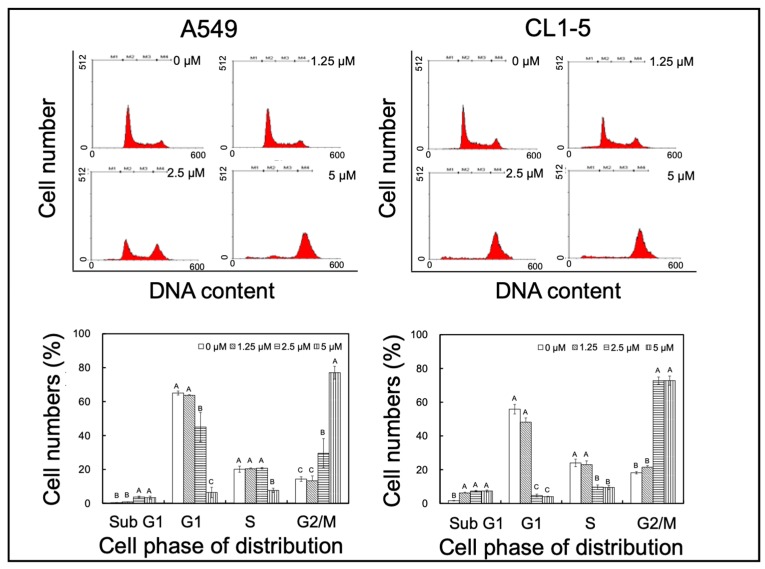
Effects of yatein treatment for 24 h with different concentrations on cell-cycle progression in A549 and CL1-5 cells. The results represent the mean ± SD (*n* = 3). Different letters indicate significant differences among each group in A549 and CL1-5 cells (*p* < 0.05).

**Figure 2:**
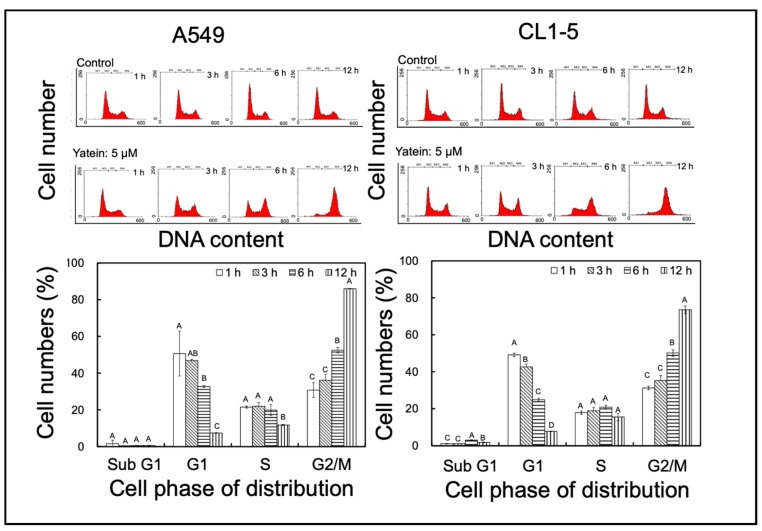
Effect kinetics of 5 μM yatein treatment on cell-cycle progression in A549 and CL1-5 cells. The results represent the mean ± SD (*n* = 3). Different letters indicate significant differences among each group in A549 and CL1-5 cells (*p* < 0.05).

**Figure 3:**
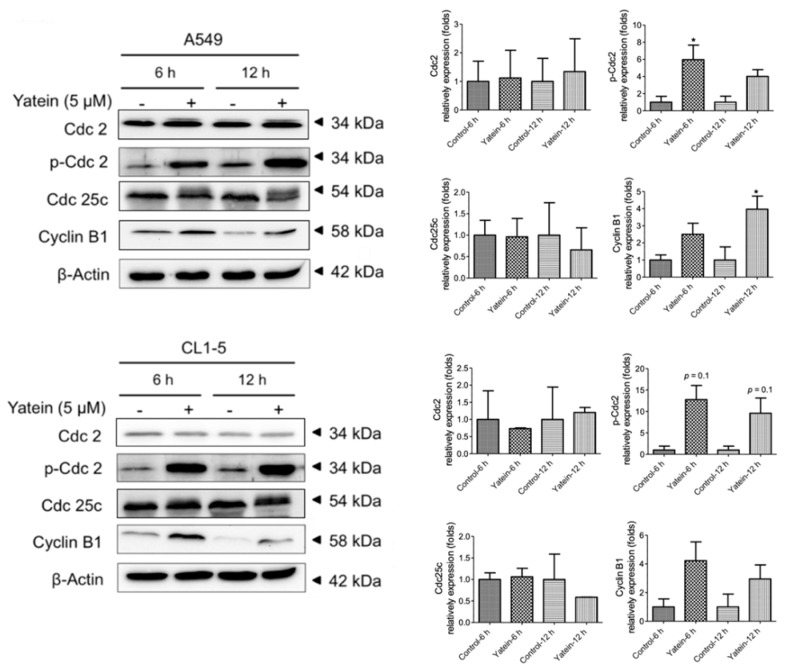
Expression of cell-cycle regulatory proteins in A549 and CL1-5 cells after yatein treatment (5 μM) for 6 h and 12 h. The bands were analyzed using the ImageJ software and normalized to β-actin expression. All data presented are representative of three independent experiments. The quantifications represent the mean ± SEM (*n* = 2‒3). * indicates a significant difference compared with the control group (*p* < 0.05).

To elucidate the mechanism underlying the anti-lung adenocarcinoma effects of yatein, cell-cycle distribution was analyzed in the yatein-treated A549 and CL1-5 cells. We found that 5 μM yatein treatment induced cell-cycle arrest at G_2_/M phase in both cell lines (Figure 1). We further analyzed the kinetics of the effects of yatein on A549 and CL1-5 cells through flow cytometry (Figure 2). Compared with untreated cells, we found that more cells entered the G_2_/M phase at 6 and 12 h after yatein treatment in both cell types. Next, we evaluated the effects of yatein on G_2_/M arrest-related protein expression using western blot analysis (Figure 3). To this end, A549 and CL1-5 cells were treated with 5 μM yatein for 6 and 12 h, and the expression of Cdc2, Cdc25c, and cyclin B1 was analyzed (Figure 3). Cdc2, Cdc25c, and cyclin B1 are key regulators of the cell cycle (particularly in the G_2_/M phase). Our results revealed that 6 and 12 h of yatein treatment upregulated cyclin B1, but not Cdc2 and Cdc25c, expression in A549 and CL1-5 cells. However, yatein treatment showed an increasing trend of Cdc2 phosphorylation in both cell types. Notably, yatein-induced Cdc2 phosphorylation was higher at 6 h than at 12 h in both the cell types, indicating that Cdc2 was involved in G_2_/M phase regulation in A549 and CL1-5 cells at an early stage.

### 2.2. Yatein Induces DNA Damage through Activation of the ATM/ATR Pathway in Human A549 and CL1-5 Cells

**Figure 4:**
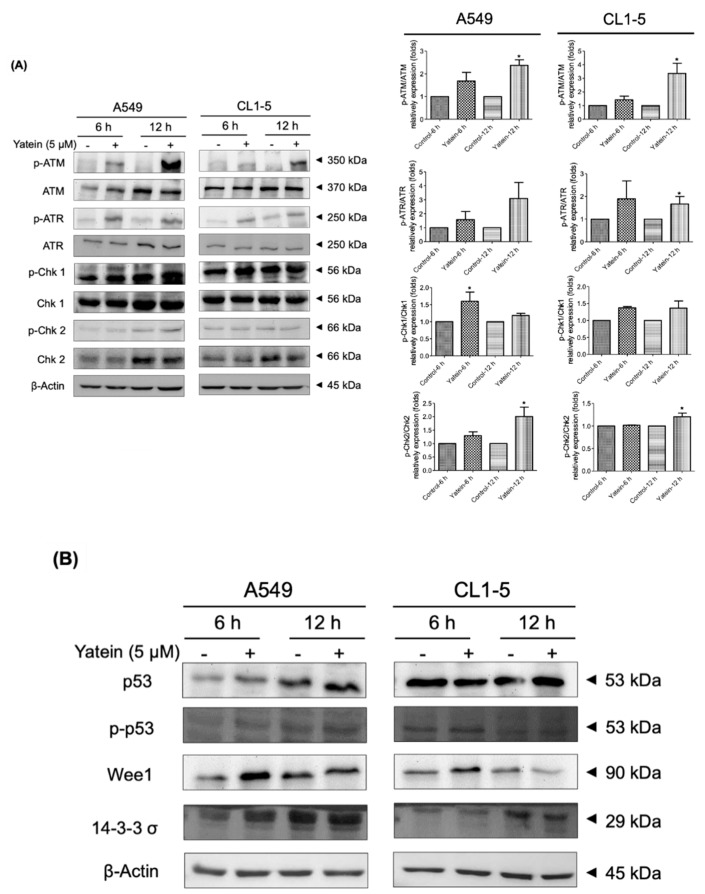
Expression of ATM, ATR, Chk1, and Chk2 (**A**), and p53 related proteins (**B**) in A549 and CL1-5 cells after yatein treatment (5 μM) for 6 and 12 h. All data presented are representative of two to three independent experiments. The quantifications represent the mean ± SEM (*n* = 2–3). * indicates significant differences compared with the control group in A549 and CL1-5 cells (*p* < 0.05).

DNA damage induces cell-cycle arrest and apoptosis in cancer cells [16]. The ATM/ATR pathway is related to DNA damage process. To address whether yatein induced DNA damage in cells, we examined the effects of yatein treatment on the ATM/ATR pathway. We found that yatein treatment showed an increasing trend of ATM and ATR phosphorylation level in A549 and CL1-5 cells for 6 h and 12 h treatments. However, ATM and ATR expression were not affected (Figure 4A). We next evaluated the expression and phosphorylation of Chk1 and Chk2 in A549 and CL1-5 cells after yatein treatment for 6 and 12 h. Our results revealed that yatein treatment showed an increasing trend of Chk1 and Chk2 phosphorylation level in A549 and CL1-5 cells. These results suggested that yatein induced DNA damage and altered cell-cycle distribution by activating the ATM/Chk2 and ATR/Chk1 DNA repair pathways in A549 and CL1-5 cells. In addition to the activation of the ATM/ATR pathway, we found that yatein treatment affected p53, Wee1, and 14-3-3 σ expression in the A549 cells (Figure 4B). Conversely, yatein did not upregulate the p53 and 14-3-3 σ expression in CL1-5 cells. However, Wee1 expression was increased after yatein treatment for 6 h in CL1-5 cells. These results indicated that the p53 pathway might play an additional role in yatein-induced growth inhibition in A549 cells but not in CL1-5 cells.

### 2.3. Yatein Influences Microtubule Dynamics in Human A549 and CL1-5 Cells

**Figure 5:**
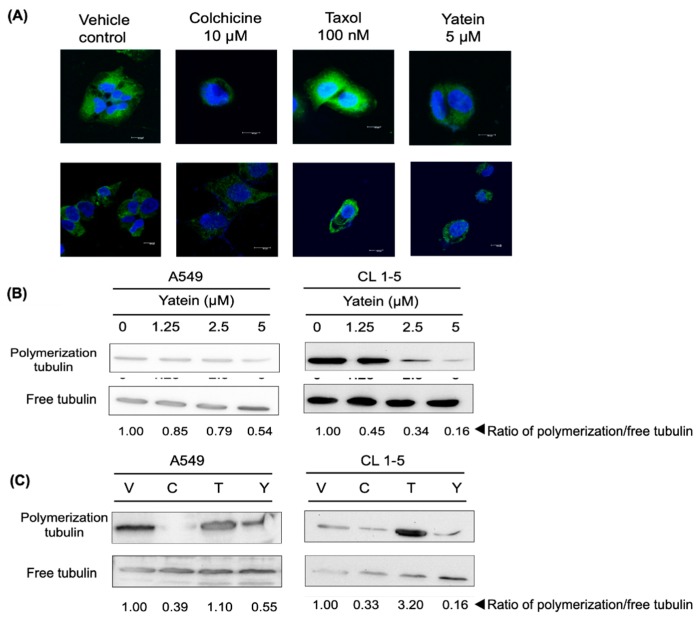
(**A**) Confocal images of tubulin expression of A549 (upper) and CL1-5 (lower) cells after yatein, colchicine, and taxol treatment for 6 h. Scale bar = 100 μm. (**B**) Western blot analysis of tubulin expression of A549 and CL1-5 cells after treatment with various concentrations of yatein for 24 h. (**C**) Western blot analysis of tubulin expression of A549 and CL1-5 cells after yatein and positive control treatment for 24 h. V: Vehicle control, C: 10 μM colchicine, T: 100 nM taxol, Y: 5 μM yatein. The quantitative values are the ratio of polymerization tubulin/free tubulin in different treatment groups.

Apart from DNA damage, inhibition of microtubule dynamics is another mechanism that induces G_2_/M cell-cycle arrest. For instance, taxol, a well-known anticancer drug, arrests cells in the G_2_/M phase by disrupting microtubule polymerization [17]. We assessed microtubule dynamics in yatein-treated A549 and CL1-5 cells using confocal microscopy and western blot analysis. Our confocal microscopy results showed that 5 μM yatein treatment caused a diffusion of green fluorescence (tubulin) in the yatein-treated A549 and CL1-5 cells compared with control cells within 6 h (Figure 5A). In addition, as shown in Figure 5B, yatein decreased tubulin polymerization in a dose-dependent manner in both A549 and CL1-5 cells after 24 h treatment. These results revealed that the effects of yatein on microtubule dynamics were similar to those of colchicine, a microtubule-depolymerizing agent (Figure 5C). Consistently, yatein-treated cells exhibited a similar pattern of tubulin distribution to that of colchicine-treated cells. By contrast, taxol-treated cells demonstrated a higher level of tubulin polymerization compared with the untreated cells. Taken together, our results indicated that yatein and colchicine affected microtubule dynamics by inhibiting tubulin polymerization.

### 2.4. Yatein Exhibits In Vivo Antitumor Effects in a Xenograft Mouse Model 

**Figure 6:**
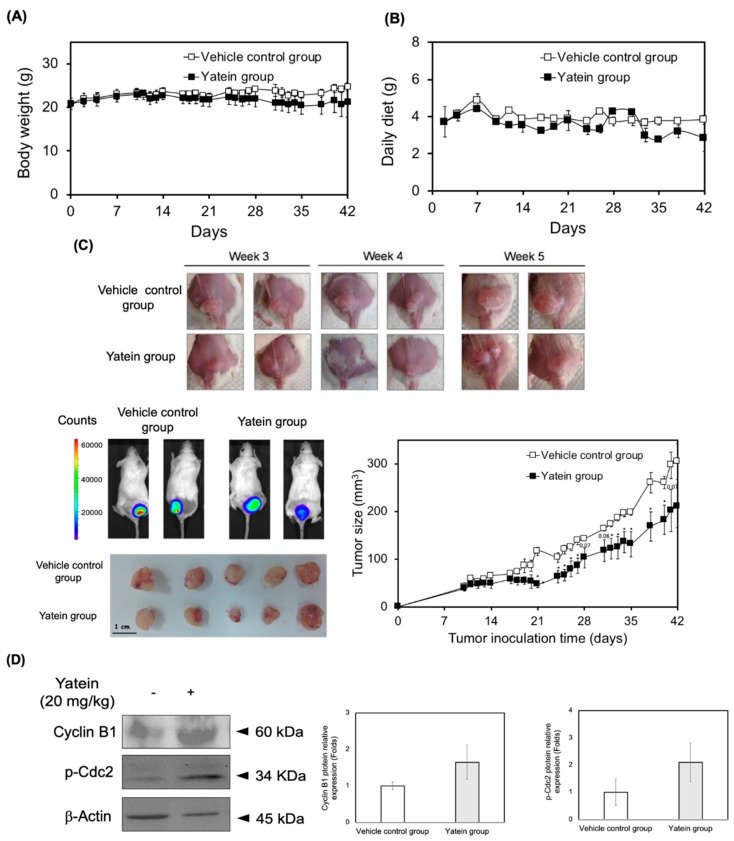
In vivo antitumor effects of yatein. (**A**) Body weight, (**B**) daily diet intake, and (**C**) tumor size and IVIS images of A549-luc cell xenograft mice treated with vehicle or 20 mg/kg of yatein during the experimental period. The results represent the mean ± SEM (*n* = 5). (**D**) Western blot analysis of tumor tissue from the A549-luc xenograft mice (values were mean ± SEM, *n* = 3).

To validate the growth inhibitory effects of yatein in an in vivo context, we applied a xenograft mouse model using A549 cells. To this end, NOD/SCID mice were inoculated with A549-luc cells for 10 days and were then given 20 mg/kg yatein (i.p.) five times per week for 42 days. Animal body weight, food intake, and tumor growth were monitored and quantified during the experiment. Our results showed that the body weight and food intake of the control and yatein-treated mice did not differ (Figure 6A,B), suggesting that yatein was well tolerated in the mice. We found that the tumor volumes of the control mice (41.3 mm^3^) and yatein-treated mice (38.5 mm^3^) were similar in the first 14 days (Figure 6C). However, within the 21‒42 days (end of experiment) time window, tumor growth in the yatein-treated mice was significantly slower compared with the control mice (*p* < 0.05). Consistently, In Vivo Imaging System (IVIS) analysis showed that the tumor tissue luminescence in the yatein-treated group (16.5 × 10^5^ photons/s) was lower than that in the vehicle control group (24.0 × 10^5^ photons/s). To confirm that yatein exhibited the same antitumor mechanisms in vivo as observed in vitro, we evaluated cyclin B1 expression and Cdc2 phosphorylation in the tumor tissue in the vehicle control and yatein-treated groups (Figure 6D). We found that both cyclin B1 expression and Cdc2 phosphorylation moderately increased in the yatein-treated group compared with the control group (Figure 6D). Taken together, these results suggested that the induction of cyclin B/Cdc2 complex expression and activation were associated with the antitumor effects of yatein in vivo.

## 3. Discussion

According to our previous study [15], the IC_50_ values for the 24 h yatein treatment in the A549 and CL1-5 cells were 10.0 and 2.1 μM, respectively. In addition, yatein exhibited excellent growth inhibitory effect in the A549 (IC_50_ values = 3.5 μM) and CL1-5 cells (IC_50_ values = 1.9 μM) after 72 h treatment. Interestingly, yatein was unable to inhibit human nasopharyngeal carcinoma (HONE-1) and human gastric cancer (NUGC) cells growth at a concentration of 50 μg/mL (≅ 125 μM) [10]. In the present study, we found that yatein induced G_2_/M cell-cycle arrest in A549 and CL1-5 cells at the first 24 h treatment. Additionally, treatment with yatein for 48 h induced a dose-dependent increase in both early and late stage apoptosis in the A549 and CL1-5 cells [15]. As previously reported, some lignan compounds can inhibit cancer cell growth by inducing cell-cycle arrest. For example, vitexin compound-1 (VB1), a lignan isolated from a plant used in traditional Chinese medicine, namely *Vitex negundo*, inhibits the growth of MDA-MB-435 and SMMC-7721 cells by inducing G_2_/M phase cell-cycle arrest after 24 h treatment at a concentration of 10 μM [18]. Similarly, benozofuran lignan shows a dose- and time-dependent induction of G_2_/M cell-cycle arrest in Jurkat T-cells [19]. Additionally, the mechanism underlying the effects of yatein on cell-cycle regulation was examined in the present study. Cyclin-dependent kinases (CDKs) collaborate with specific cyclins to tightly regulate cell cycle and cell division. Thus, the CDK/cyclin complex plays a key role in cell-cycle progression [20]. Our results indicated that yatein treatment showed an increasing trend of cyclin B1 expression and Cdc2 phosphorylation level. Aberrant activation of cyclin B1/p-Cdc2 activity is closely associated with mitotic catastrophe. Mitotic catastrophe is a type of cell death that results from mitotic regulation dysfunctions and can be induced by chemical and physical stresses [21]. Liu et al. [22] reported that expression of cyclin B1 and level of Cdc2 phosphorylation were increased in malignant glioma cells treated with a synthetic quinazolinone analog (MJ-66), suggesting that MJ-66 induced G_2_/M arrest and mitotic catastrophe in malignant glioma cells. Subramaniam et al. [23] reported that curcumin-induced mitotic catastrophe coupled with increased expression of cyclin B1 and Cdc-2 in MiaPaCa-2 cells. Therefore, we can speculate the cytotoxic effects of yatein on A549 and CL1-5 cells might be partially due to the induction of mitotic catastrophe. The correlation between yatein-induced cell death and mitotic catastrophe still need to be investigated in a future study.

DNA damage is vital in the regulation of cell cycle and apoptosis. In this study, we found that yatein induced cell-cycle arrest by inducing DNA damage in A549 and CL1-5 cells. In general, ATM and ATR kinases are the initiating kinases of the DNA damage response (DDR) signaling pathway that is activated by DNA damage or DNA replication stress [24]. ATM is activated by DNA double-strand breaks, while ATR is activated by various factors related to DNA damage [25]. According to previous studies, several natural products, including berberine, curcumin, and sinularin, induced DNA damage and G_2_/M arrest, but not S phase arrest, in various cancer cells [26,27,28]. The results revealed that these natural products induced DNA damage and arrested cells at G_2_/M phase. At the same time, the ATM pathway was up-regulated [26,27,28]. After ATM activation, several proteins involved in the regulation of ATM on DNA repair, cell-cycle arrest, apoptosis, and other downstream processes, such as Brca1, Chk2, and p53, are phosphorylated [29]. Our results revealed that yatein induced the expression of ATR/Chk1 and ATM/Chk2 in A549 cells. Notably, only ATR/Chk1 expression was upregulated after yatein treatment in CL1-5 cells. We found that the expression and phosphorylation of p53, Wee1, and 14-3-3 σ expression were induced after yatein treatment in A549 cells, suggesting that yatein activated p53-mediated signaling pathway to inhibit growth in A549 cells. Yatein did not affect the expression of p53 and its related proteins in CL1-5 cells; this was because the CL1-5 cell line carries a p53 mutation, which has been implicated in more than 50% of all cancer cases [30]. The p53 mutation causes a defective G_1_ checkpoint in cancer cells that results in increased DNA damage at the G_2_ checkpoint compared with noncancer cells. Wee1 is a key protein that is closely associated with G_2_ checkpoint abrogation and mitotic catastrophe [30,31]. Accordingly, we found that yatein treatment increased Wee1 expression in CL1-5 cells, indicating that Wee1 played a crucial role in the regulation of G_2_/M cell-cycle distribution in CL1-5 cells in the context of yatein treatment. On the other hand, we also found yatein is able to induce ROS production in A549 and CL1-5 cells in our previous study [15], suggesting that yatein may induce the oxidative DNA damage. Moreover, our previous results also revealed that the CL1-5 cells were more sensitive for the ROS production than the A549 cells after yatein treatment [15].

Additionally, we showed that yatein affected the microtubule assembly in both A549 and CL1-5 cells after 6 h treatment (Figure 5A) and 24 h treatment (Figure 5B and 5C). The induction of mitotic arrest is associated with dysfunctional microtubule dynamics [32]. Microtubules play vital roles in cell proliferation, trafficking, signaling, and migration [33]. Considering the importance of microtubule dynamics, several small microtubule-targeting molecules have been designed and used as anticancer drugs [30]. Tubulin has three major binding sites: taxane, vinca, and colchicine domains. In this study, we found that yatein exhibited similar effects to that of colchicine, implying that yatein inhibited tubulin polymerization. These results suggested that tubulin polymerization inhibition partially contributed to the ability of yatein to arrest A549 and CL1-5 cells at the G_2_/M phase.

**Figure 7:**
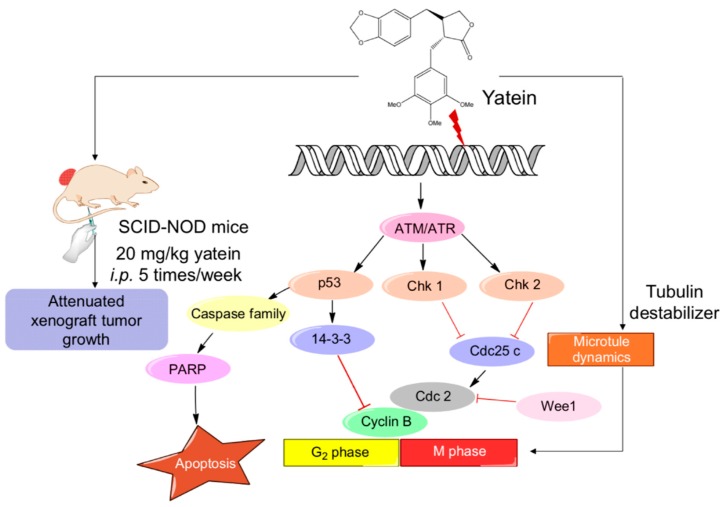
Proposed mechanism of the inhibitory effects of yatein on lung adenocarcinoma cells.

The in vivo growth inhibitory effects of yatein on A549 cells were elucidated in this study. Our results revealed that yatein treatment significantly suppressed tumor growth in mice without affecting their body weight and food intake. Additionally, yatein treatment increased cyclin B1 expression and Cdc2 phosphorylation in vivo, indicating that the induction of mitotic catastrophe was involved in the anticancer mechanism of yatein in vivo. Taken together, we found that yatein affected cell-cycle progression and microtubule dynamics, induced DDR, and exhibited anticancer properties in vivo (Figure 7).

## 4. Materials and Methods

### 4.1. Preparation of Yatein

The phytocompound, yatein, was isolated from *C. formosana* leaves extracts. In brief, *C. formosana* leaves were extracted using methanol at room temperature (RT) for one week (twice) to obtain a methanolic extract. The dried samples were further divided to *n*-hexane, ethyl acetate (EtOAc), *n*-butanol, and H_2_O fractions using liquid–liquid partition. The *n*-hexane fraction was further fractionated into ten subfractions using normal phase column chromatography (Geduran Si-60, Merck, Darmstadt, Germany). Yatein was isolated and purified from the subfraction 4 by semipreparative high-performance liquid chromatography using a PU-2080 pump (Jasco, Tokyo, Japan) equipped with an RI-2031 detector (Jasco) and a 5 μm Luna silica column (250 mm × 10.0 mm internal diameter; Phenomenex, Torrance, CA, USA). The mobile phase consisted of 30% of EtOAc and 70% of *n*-hexane (*v*/*v*), and the flow rate was 4 mL/min. The retention time of yatein in HPLC analysis was 18.0 min. The purity and the structure elucidation of yatein was conducted by ^1^H and ^13^C NMR, and all spectrum data were consistent with literature [34].

### 4.2. Cell Culture

Human A549 cell line was purchased from Bioresource Collection and Research Center (BCRC 60124) and cultured in RPMI-1640 (Gibco, Gran Island, NY, USA) supplemented with 10% (*v*/*v*) fetal bovine serum (Gibco) and 1% (*v*/*v*) antibiotic–antimycotic agent (Gibco). CL1-5 cell line was provided by Dr. Jeremy J.-W. Chen (National Chung Hsing University, Taichung, Taiwan) and cultured in Dulbecco’s modified Eagle’s medium (Gibco) supplemented with 10% (*v*/*v*) fetal bovine serum (Gibco) and 1% (*v*/*v*) antibiotic–antimycotic agent (Gibco). The cells were incubated in a 37 °C humidified incubator containing 5% CO_2_.

### 4.3. Cell-Cycle Distribution Analysis

The A549 and CL1-5 cells were seeded onto a 6-well plate at a density of 1 × 10^5^ cells/well and incubated overnight. The cells were then treated with various concentrations of the test samples for the indicated durations. After treatment, the cells were collected and mixed with ice-cold 75% ethanol and then fixed overnight at −20 °C. Then, the cells were washed twice using phosphate-buffered saline (PBS), and then incubated with 200 μL of propidium iodide (PI, BD Bioscience, Franklin Lakes, NJ, USA) solution (containing 1 mg/mL PI, 2 mg/mL RNase A, and 0.5% Triton X-100) for 30 min at RT in the dark. After 30 min, the PI-stained cells were immediately analyzed using a flow cytometer (Accuri 5, Accuri Cytometers, Inc., Ann Arbor, MI, USA). The data were analyzed using the C6 Accuri system software (Accuri Cytometers, Inc.).

### 4.4. Isolation of Microtubule Proteins

The A549 and CL1-5 cells (1 × 10^6^ cells) were seeded onto culture dishes (10 cm) overnight and treated with 5 μM yatein. The cells were harvested through trypsinization and washed with PBS. After centrifugation, the supernatant was removed and the cell pellets were mixed with 200 μL of microtubule stabilizing buffer (1 mM MgCl_2_, 2 mM Tris-HCl, 2 mM EGTA, and 0.5% Triton-100 in ddH_2_O) and incubated at RT for 20 min. Subsequently, the mixture was centrifuged at 12,000× *g* for 10 min (4 °C) to obtain the supernatant (monomer tubulin fraction). The remaining cell pellets were washed using the microtubule stabilizing buffer and lysed in a radioimmunoprecipitation assay buffer containing 10% proteinase inhibitor and 10% phosphatase inhibitor (Sigma-Aldrich, St. Louis, MO, USA) at 4 °C for 30 min to obtain the polymer tubulin fraction. The monomer tubulin and polymer tubulin fractions were transferred into microtubes (1.5 mL) and stored at −20 °C until further analysis.

### 4.5. Western Blot Analysis

The expression of proteins in cells were determined using western blot analysis as previously reported [15]. In this study, the primary antibodies were anti-14-3-3 σ (Santa Cruz, Dallas, TX, USA), anti-β-actin (Santa Cruz), anti-ATM (Santa Cruz), anti-ATR (Santa Cruz), anti-Cdc2 (Abcam, Cambridge, MA, USA), anti-Cdc25c (Santa Cruz), anti-Chk1 (Santa Cruz), anti-Chk2 (Santa Cruz), anti-cyclin B1 (Abcam), anti-p53 (Cell Signaling Technology Inc, Danvers, MA, USA), anti-phospho-p53 Ser 15 (Cell Signaling Technology Inc), anti-phospho-ATM (Santa Cruz), anti-phospho-ATR (Santa Cruz), anti-phospho-Cdc2 (Santa Cruz), anti-phospho-Chk1 (Santa Cruz), anti-phospho-Chk2 (Santa Cruz), anti-β-tubulin (Abcam), and anti-Wee1 (Santa Cruz) antibodies. An enhanced chemiluminescence (ECL, Sigma-Aldrich) system was used for developing signals of the blots, which were analyzed using a LAS3000 system (Fujifilm, Tokyo, Japan).

### 4.6. Immunofluorescence

The A549 and CL1-5 cells (1 × 10^5^ cells) were seeded on a chamber slide for 24 h and treated with colchicine (10 μM), taxol (100 nM), and yatein (5 μM) for 6 h. The cells were washed with PBS and fixed with 1% paraformaldehyde (Sigma-Aldrich) at RT for 30 min. The paraformaldehyde was removed, and the cells were washed thrice with PBS. Next, the cells were blocked using 1% BSA in PBS at 4 °C overnight. After they were washed with PBS, the cells were incubated with the anti-β-tubulin antibody (1:200 in 1% BSA solution) at 4 °C overnight. After washing with PBS, an anti-rabbit-FITC antibody was added to the cells and then incubated overnight. Then, the antibody was rinsed off and the cells were washed thrice using PBS. 4,6-Diamidino-2-phenylindole was added and the cells were incubated for 15 min at RT in the dark. A Leica TCS SP2 confocal spectral microscope (Buffalo, NY, USA) was used to observe immunofluorescence staining of microtubule dynamics.

### 4.7. In Vivo Antitumor Activity

The procedures involving animals were performed according to the guidelines of the Institutional Animal Care and Use Committee of National Chung Hsing University (IACUC no. 107-127). The A549-luc cells were mixed with Matrigel (Sigma-Aldrich) at a 1:1 ratio. The cells were injected subcutaneously into the back of nonobese diabetic and severe combined immunodeficiency (NOD/SCID) mice (male, 6–8 weeks old) at a density of 3.5 × 10^6^ cells/mouse. Tumors were allowed to grow for 10 days and were then treated with an intraperitoneal (i.p.) injection of either 0.5% DMSO in ddH_2_O to the mice in the vehicle control group (*n* = 5) or 20 mg/kg of yatein (dissolved in 0.5% DMSO in ddH_2_O) to the mice in the yatein group (*n* = 5). The tumor-bearing mice were sacrificed after 42 days. Tumor volume was measured five times/week and calculated using the following formula: Length × width × thickness × 0.5 (mm^3^). An IVIS (Caliper lifescience IVIS Spectrum CT) was used to analyze the luminescence of tumor tissue.

### 4.8. Statistical Analysis

Data are expressed as mean ± standard deviation (SD) or mean ± standard error of the mean (SEM). Statistical analysis was performed using the shuffle test or non-parametric Kruskal-Wallis test with Dunn’s post hoc tests. A *p* of < 0.05 was considered statistically significant.

## 5. Conclusions

The present study demonstrated that yatein suppressed lung adenocarcinoma cancer cells growth by inducing cell-cycle arrest, mitotic catastrophe, and microtubule depolymerization. Yatein induced G_2_/M arrest by upregulating the expression of cyclin B1 and Cdc2 phosphorylation in lung adenocarcinoma cancer cells. In addition, mitotic catastrophe and microtubule depolymerization were involved in yatein-mediated lung adenocarcinoma cancer cells growth inhibition. Furthermore, we confirmed the in vivo antitumor effects of yatein using a xenograft mouse model. These findings provide novel insights into the in vitro and in vivo antitumor efficacy of yatein and demonstrate the potential of this phytocompound as an anticancer lead compound for lung adenocarcinoma cancer treatment.
